# RNA synthesis is modulated by G-quadruplex formation in Hepatitis C virus negative RNA strand

**DOI:** 10.1038/s41598-018-26582-3

**Published:** 2018-05-25

**Authors:** Chloé Jaubert, Amina Bedrat, Laura Bartolucci, Carmelo Di Primo, Michel Ventura, Jean-Louis Mergny, Samir Amrane, Marie-Line Andreola

**Affiliations:** 1 Univ Bordeaux, CNRS UMR5234, MFP laboratory, F-33000 Bordeaux, France; 2 Univ Bordeaux, ARNA laboratory, INSERM U1212, CNRS UMR 5320, IECB, F-33600 Pessac, France; 3 Institute of Biophysics, Academy of Sciences of the Czech Republic, 612 65 Brno, Czech Republic

**Keywords:** Virology, Structural biology

## Abstract

DNA and RNA guanine-rich oligonucleotides can form non-canonical structures called G-quadruplexes or “G4” that are based on the stacking of G-quartets. The role of DNA and RNA G4 is documented in eukaryotic cells and in pathogens such as viruses. Yet, G4 have been identified only in a few RNA viruses, including the Flaviviridae family. In this study, we analysed the last 157 nucleotides at the 3′end of the HCV (−) strand. This sequence is known to be the minimal sequence required for an efficient RNA replication. Using bioinformatics and biophysics, we identified a highly conserved G4-prone sequence located in the stem-loop IIy’ of the negative strand. We also showed that the formation of this G-quadruplex inhibits the *in vitro* RNA synthesis by the RdRp. Furthermore, Phen-DC3, a specific G-quadruplex binder, is able to inhibit HCV viral replication in cells in conditions where no cytotoxicity was measured. Considering that this domain of the negative RNA strand is well conserved among HCV genotypes, G4 ligands could be of interest for new antiviral therapies.

## Introduction

DNA and RNA guanine-rich regions can fold into non-canonical secondary structures called G-quadruplexes or “G4”. These structures result from the planar association of four guanines connected through hydrogen bonding. These G-quartets stack on top of each other and are stabilized by monovalent cations such as sodium or potassium. Since the 1960s, when the structural basis of the G-tetrads was elucidated, numerous works have described their implication in biological regulatory mechanisms. Control of telomeric functions, DNA replication, gene expression, genome stability, translation regulation or mRNA processing can involve DNA or RNA G-quadruplexes^1^. These structures are also present in pathogens and can control their virulence^2,3^. In addition to bacteria and protozoa, several DNA viruses are concerned (for a review^3^). The Epstein–Barr virus encodes a genome maintenance protein (nuclear antigen 1, EBNA1) that binds G-rich sequences at the viral replication origin, recruits the replication complex and is involved in metaphase chromosome attachment. This interaction ensures the genome maintenance throughout mitosis^4^. The human papillomaviruses (HPV) genome also contains G-quadruplexes that can carry out regulatory functions^5^. In SV40, the last regulatory region contains six GC boxes (GGGCGG) in close proximity involved in a quadruplex structure that constitutes a binding motif for SP1^6^. The early transcription of SV40 is also controlled by G4 structures. The HSV-1 genome displays multiple clusters of repeated sequences forming very stable G-quadruplexes that are involved in the viral DNA replication^7^. Beside these DNA viruses, there are very few descriptions of G-quadruplexes in RNA viruses. HIV-1 is the first one in which RNA G4 structures have been shown to be essential in the replication cycle. They are needed for genome dimerization^8^ and for capsid attachment^9^. A conserved G-rich region from HIV-1 promoter was shown to form G4 structures^10^ and being involved in the regulation of viral replication^11^. Very recently, G-rich sequences were identified and analyzed in the genome of Ebola virus and Marburg virus^12,13^. A recent study has also revealed the first evidence of G-rich sequences to be found in the negative-sense RNA of Zaire ebolavirus L gene and likely involved in the L gene expression^14^. Finally, several potential G4 forming sequences were identified in Zika genome, belonging to the Flaviviridae family^15^. Their role in the viral replication, if any, still has to be determined.

**Figure 1:**
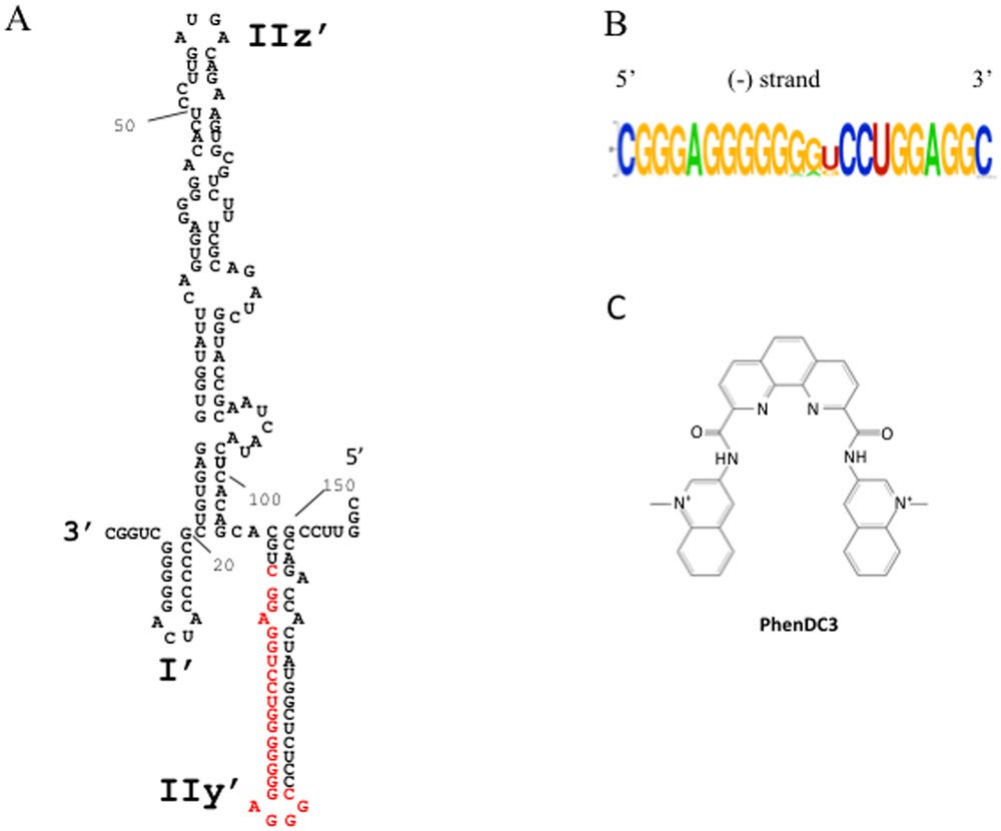
(**A**) Predicted secondary structure of the 3′end 157 nucleotides of the HCV (−)RNA according to Smith *et al*.^21^. The putative G4 forming sequence used for the biophysical experiments (HCV110-131) is highlighted in red. (**B**) Logo representation of the detected putative G4 sequence by G4Hunter algorithm (WebLogo application). (**C**) Chemical structure of PhenDC3 G4 ligand.

Hepatitis C virus (HCV), the causative agent of hepatitis C, a liver disease, also belongs to the Flaviviridae family. Its plus-strand RNA genome of 9600 nt is flanked by 5′ and 3′ untranslated regions (UTRs) that fold in several stem-loop structures engaged in short or long-range RNA-RNA tertiary interactions^16,17^. These interactions are essential for viral replication^18–20^. The HCV genome encodes a unique polyprotein, which is cleaved into three structural proteins (C, E1, E2), and seven non-structural proteins (p7, NS2, NS3, NS4a, NS4b, NS5a and NS5b). The latter is an RNA-dependent RNA polymerase (RdRp) that, associated to cellular and viral non-structural proteins in a replication complex (RC), ensures the RNA synthesis. Plus-strand genomic RNA ((+)RNA) is copied into a negative strand ((−)RNA) that is used as template for the synthesis of new (+)RNAs. The RC binds to the 3′-end RNA of each strand to ensure the synthesis of a complete genome. As a result, the 3′-end of the (−)RNA is used by the RC to initiate the RNA synthesis of the (+)strand. However, the intimate mechanism involved in the initiation of the RNA synthesis is not known. The secondary structure of the 3′end of the (−)RNA was established from RNA footprinting experiments (Fig. 1A)^21,22^. Some structural elements of this structure were shown to be implicated in the initiation of RNA synthesis^23–25^. Furthermore, it has been shown that the last 157 nucleotides at the 3′-end of the HCV (−)strand constitute the minimal sequence required for efficient RNA replication^26,27^.

Recently a G-rich region located in the core coding sequence on the genomic (+)strand RNA of HCV was shown to adopt a G4 structure^28^. In this work, we identified another G-rich region in the stem-loop IIy’ of the 3′-end of the negative strand of HCV. Using bioinformatics, biophysics, biochemical and cellular experiments, we show that this G-rich sequence is able to fold into a G4. *In vitro* RNA synthesis by the RdRp is affected by the formation of this quadruplex. Furthermore, we show that Phen-DC3, a known G-quadruplex ligand, inhibits HCV viral replication in cells. As this region of the (−)strand RNA of HCV is highly conserved, targeting the G4 structure by ligands might be a new therapeutic pathway.

## Material and Methods

### Oligonucleotides synthesis and samples preparation

Oligonucleotides were purchased from Eurogentec (Seraing, Belgium) with RP cartridge gold purification. Oligonucleotide strand concentrations were determined by measuring absorbance at 260 nm (SAFAS UVmc2 double-beam spectrophotometer (Monte Carlo, Monaco) using the extinction coefficients provided by the manufacturer.

### Cells and viruses

Huh7 cells were grown in Dulbecco’s modified Eagle medium supplemented with 10% fetal calf serum and gentamicin (50 µg/mL) at 37 °C in a 5% CO_2_ atmosphere. The fully replicative whole HCV genome (LMTV, JFH-1 backbone, Genbank HG948568) was obtained by gene synthesis (Eurofins MWG/Operon, Ebersberg, Germany) and cloned into pUC19 (pUC/LMTV). The gene encoding Gaussia luciferase (GLuc) ending by 60 nt that coded for the FMDV 2 A protein, was obtained by gene synthesis and was further introduced between the p7 and NS2 genes in position 2779 (pUC/LMTV-GLuc) as described in^28^.

### UV melting and thermal difference spectra (TDS)

A SAFAS UVmc2 double-beam spectrophotometer (Monte Carlo, Monaco) equipped with a 10-cells holder regulated by a Peltier controller was used to perform the UV melting experiments. Oligonucleotide strand concentrations ranged between 3.3 and 7.2 µM. The melting curves at 295 nm were recorded both ways between 95 °C and 5 °C with a temperature gradient of 0.2 °C/min^29^. TDS were calculated by subtracting the UV spectrum at 5 °C from the one at 95 °C^30^.

### Circular dichroism (CD) spectroscopy

CD spectra were measured at 20 °C between 220 and 335 nm (0.5 nm data pitch) in 1 cm path-length quartz cells using a Jasco J-815 instrument. Each spectrum was the average of four scans recorded at 50 nm/min (2 nm bandwidth and 1 s data integration time). The oligonucleotide concentration was 2.5 µM. CD melting curves were obtained by measuring the CD signals from low temperature to high temperature with 1 °C increments and a temperature gradient of 0.3 °C/min. The ellipticity was then plotted at 263 nm against temperature.

### NMR spectroscopy

NMR experiments were performed on a 700 MHz Bruker spectrometer equipped with a TXI probe. ^1^H 1D NMR experiments were acquired using a pulse sequence with Spin-Echo Water Suppression. The oligonucleotides were dissolved in a 20 mM potassium phosphate buffer, pH 7 at 20 °C, and 70 mM KCl at a concentration of 0.15 mM.

### FRET melting assay

The ligand-induced thermal stabilisations (∆T_1/2_) were determined from FRET melting experiments carried out in 96-well plates on a Stratagene Mx3005P real-time PCR equipment, as previously described^30^. The excitation and detection wavelengths were set to 492 and 516 nm, respectively. After an initial stabilisation at 25 °C for 5 min, the temperature was increased by a 1 °C step every minute until 95 °C. When the RNA sequence is in the G-quadruplex folded state, FRET occurs between the two fluorophores while denaturation of the G-quadruplex structure upon heating abolishes the FRET process. The fluorescently labelled HCV110-131 sequence (f-HCV110-131-t 5′-FAM-CGGGAGGGGGGGUCCUGGAGGC-Tamra-3′) was dissolved to a 0.1 mM stock concentration and stored at −20 °C. The stock concentrations were determined from the absorbance at 260 nm measured on a SAFAS UVmc2 double-beam spectrophotometer (Monte Carlo, Monaco) (according to the molar extinction coefficient mentioned on the corresponding technical data sheet). The melting experiments were performed at a final 0.2 μM strand concentration of labeled oligonucleotide with 0.4 μM of ligand Phen-DC3. The experiments were carried out in a 10 mM lithium cacodylate buffer (pH 7.2 at 20 °C) containing either 10 mM KCl or 90 mM LiCl.

### *In vitro* transcription

The pGEM9Zf(–) containing the 341 nucleotides of the 5′UTR of HCV (H77 strain) was used for *in vitro* transcription to produce the HCV (−)RNA 3′-end (157nt)^22^. PCR was performed with the GoTaq® Flexi DNA Polymerase (Promega) with primers designed to introduce a T7 RNA polymerase promoter in the correct orientation. The primers sequences are Stop_157: GCCAGCCCCCTGATGGGGGCGACAC, T7_157_WT: TAATACGACTCACTATAGGTTCCGCAGACCACTATGGC or T7_157_MutG4: TAATACGACTCACTATAGGTTCCGCAGAGGACTATGATTTTTTTGGGAAAAAAAATCCTCCAGGCTGCACG. Control RNA 5BSL3.2 (555 nt) was produced similarly using pGEM-T/5UTR-H2AE-5BSL-3UTR vector^31^ and specific primer (Stop_555: ACTTGATCTGCAGAGAGGCCAG and T7_555: TAATACGACTCACTATAGGGCATGGACGACTGTACAAGTAATC). The template for producing the LMTV genomic RNA was obtained by cleavage of the XbaI site of pUC/LMTV-Gluc. The RNA was then obtained from 1 µg of DNA by *in vitro* transcription using the MEGAscript kit (Ambion). DNA templates were digested with DNase I for 15 min then precipitated after acidic phenol/chloroform extraction. HCV107-151 negative strand including the sequence corresponding to the inverse complementary of the T7 promoter was obtained from Eurofins Genomics SAS (GCAGCCTCCAGGACCCCCCCTCCCGGGTGTGCCATAGTGGTCTGCCCTATAG TGACTCGTATTA). One µg of HCV107-151 and 4 µg of portable promotor pT7 (CCTATAGTGACTCGTATTA) were incubated with 2 µl of the transcription buffer MEGAscript kit (Ambion) for 1 min at 95 °C and decrease to 30 °C for 30 min before adding the other components of the transcription kit and transcribed for 2 hours. DNA templates were digested with DNase I for 15 min then precipitated after acidic phenol/chloroform extraction. The purity and integrity of the RNAs were determined using capillary electrophoresis on a Bioanalyzer 2100 (Agilent).

### RNA-dependent RNA polymerase (RdRp) assay

The assay was performed in a final volume of 25 µL containing 20 mM Tris-HCl buffer (pH 7.5), 1 mM DTT, 5 mM MgCl_2_, 40 mM NaCl or KCl, 13.2 U RNasin (Promega), 0.5 mM each of the 3 NTP (ATP, CTP, GTP), 500 nM G4-ligand when indicated, 10 pmoles of RNA template, 8 pmoles of purified NS5B (purified as in^22^) and 2 µCi [^3^H]UTP (40 Ci/mmol). The reaction mixture was incubated for 10 min at 30 °C before addition of the purified NS5B for 2 h at 30 °C. The reaction was stopped by the addition of 10% trichloracetic acid (TCA) onto 20 µl of the mixture. The radioactivity incorporated into neo-synthesized RNA was then determined by scintillation counting and normalized according to the number of UTP contained in each template.

### Cell culture and LMTV infection

Huh7 cells (25000 per well) were seeded in a 96-wells plate 24 hours before infection. Serial dilutions of G4 ligand or DMSO were added on the cells. Infection was then performed using supernatant containing LMTV particles. After 48 hours of culture, the GLuc activity from 4 µl of viral supernatant diluted in 1 µl of lysis buffer (Luciferase Cell Lysis Buffer, BioLabs) was measured using 30 µL of BioLux GLuc Substrate (BioLabs) and the VariosKan apparatus (ThermoScientific). Cytotoxicity analysis was performed in similar conditions without virus and measured with the CellTiter 96® AQueous One Solution Cell Proliferation Assay System (Promega).

### G4 prediction

The G4Hunter algorithm was used to predict putative G4 structures in the genome^32^. The mean of the scored nucleic acid sequence was computed for a sliding window arbitrary set at 25 nt. Only scores above 1.0 were taken into consideration.

## Results

### G4Hunter software predicts G4 RNA formation in the 3′-end of the HCV negative strand

We used the in-house G4Hunter algorithm to search for G4 putative sequences in a FASTA alignment of 107 HCV genomes (+)RNA from the “HCV genome database” (www.hcv.lanl.gov). A G4-prone fragment of the FASTA alignment was detected. This G4 sequence is located in the stem-loop IIy’ of the 3′-end of the (−)RNA (Fig. 1A, in red). Interestingly, despite the high genetic variability of HCV^33^, this sequence is highly conserved across 106 sequences from various HCV strains (Fig. 1B and Table 1). The four blocks of Gs are always present, with only slight differences in length and base composition of the middle linker that separates the second and third G4 stretches. The sequence CGGGAGGGGGGGTCCTGGAGGC (termed HCV110-131) was the most frequently identified and occurred 65 times among the 106 sequences. In addition, 5 different motifs with G4Hunter scores ranging from 1.4 to 1.77 were found in the 42 other sequences (Table 1). According to our experience with hundreds of sequences experimentally tested in our previous work^32^, all of these sequences are very likely to form a stable G4 *in vitro*. Interestingly, Quadparser would have failed to pick them as two of the G-blocks contain only two consecutive guanines. This could explain why G4 formation in this region was overlooked using classical algorithms.

**Table 1:** G4 prone sequences detected by G4hunter. Mutations as compared to the most frequently found motif (HCV110-131) are shown in bold/italic.

Sequences	Occurrence in the alignment	G4H Score
CGGGAGGGGGGGUCCUGGAGGC (HCV110-131)	65	1.77
CGGGAGGGGGGG***G***CCUGGAGGC	21	1.95
CGGGAGGGGGG***A***UCCUGGAGGC	14	1.59
CGGGAGGGGG***A***GUCCUGGAGGC	4	1.45
CGG***A***AGGGGGGG***G***CCUGGAGGC	1	1.73
CGGGAGGGGGGG***A***CCUGGAGGC	1	1.77

### HCV110-131 sequence forms a G4 *in vitro*

**Figure 2:**
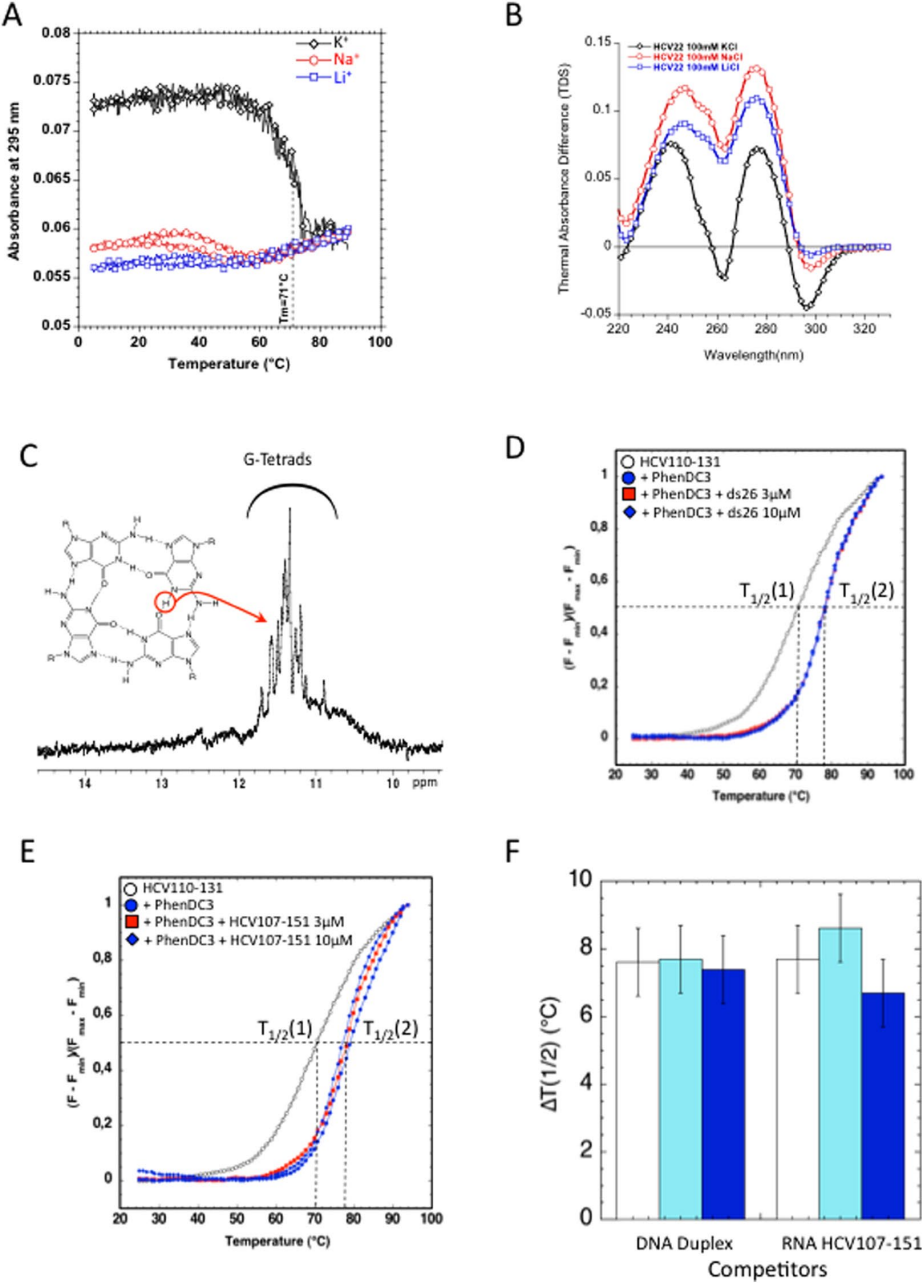
Biophysical evidence of RNA G4 formation at the 3′end of HCV negative strand. (**A**) Melting profiles from HCV110-131 5′CGGGAGGGGGGGUCCUGGAGGC3′ recorded at 295 nm in the presence of 10 mM lithium cacodylate buffer, pH 7.0 at 22 °C, containing either 100 mM of KCl, NaCl or LiCl. The HCV110-131 oligonucleotide concentration was 2.5 µM. (**B**) Thermal differential spectra recorded in the presence of 10 mM lithium cacodylate buffer, pH 7.0, containing either 100 mM of KCl, NaCl or LiCl. The HCV110-131 oligonucleotide concentration was 2.5 µM. (**C**) ^1^H, 1D NMR spectrum recorded in 20 mM potassium phosphate buffer, pH 7, containing 70 mM KCl. The oligonucleotide concentration was 100 µM. **D**,**E**,**F**. FRET-melting assay recorded for f-HCV110-131-t RNA at 0.2 µM in 10 mM lithium cacodylate pH 7.2 containing 10 mM KCl and 10 mM LiCl. (**D**) FRET-melting in absence of ligand (white circles) or in the presence of 0.4 µM of ligand (blue circles, red square, blue square). For competition 3 µM and 10 µM of ds26 were added to the solution (red square and blue square). (**E**) FRET-melting in the absence of ligand (white circles) or in the presence of 0.4 µM of ligand (blue circles, red and blue squares). For competition 3 µM and 10 µM of HCV107-151 were added to the solution (red square and blue square). (**F**) Histogram representation of the stabilisation induced by PhenDC3 in absence of competitor (white) or in the presence of 3 µM (Cyan) or 10 µM (Blue) of competitor ds26 or HCV107-151.

To assess whether the G-rich conserved sequence is able to fold into G4, an RNA fragment corresponding to the HCV110-131 region was studied using several biophysical methods. The UV-melting profiles of HCV110-131 recorded at 295 nm revealed that the melting temperature strongly depended on the nature of the cation (Fig. 2A). A highly cooperative inverted transition with a melting temperature of 71 °C was observed in the presence of 100 mM KCl. In contrast, very weak transitions were observed in the presence of sodium and lithium. This demonstrates that this oligoribonucleotide forms a stable G4 structure only in the presence of potassium. The superimposition of the heating and cooling melting curves in potassium conditions suggests that the G4 structure is intramolecular, with fast kinetics of folding and unfolding.

TDS analysis confirmed the formation of a G-quadruplex, with a clear negative peak around 295 nm obtained in the presence of potassium. In contrast, the TDS spectra recorded with sodium or lithium cations displayed low intensity peaks at the G4 characteristic 295 nm wavelength (Fig. 2B). These data suggest that only a minor proportion, if any, of the oligonucleotide is able to form a G4 in sodium and lithium conditions.

NMR analysis confirmed the formation of a G-quadruplex, as shown by the imino proton NMR spectrum of HCV110-131 in potassium (Fig. 2C). Indeed, the detection of well-resolved imino proton peaks in the 11−12 ppm region indicates the presence of Hoogsteen type Hydrogen bonds known to be involved in the formation of G-tetrads.

To further support these results, we used Phen-DC3, a well-described G4 ligand^34^, to probe the formation of G4 structures. This bisquinolinium derivative (Fig. 1C) is highly specific for G-quadruplexes; it strongly binds to the G4 structures but weakly to duplexes^35^. We evaluated the ability of Phen-DC3 to interact with HCV110-131 G4 using the classical fluorescence resonance energy transfer (FRET) melting assay^32^ with a 5′-FAM/3′-TAMRA labelled oligonucleotide (Fig. 2D,E). When the RNA G4 is in the folded state, FRET occurs between the two fluorophores while denaturation of the G4 upon heating abolishes the fluorescence energy transfer. The ligand-induced stabilisation of a folded G-quadruplex was determined by measuring the increase of the melting temperature (∆T_1/2_). In this test, a T_1/2_ of 70 °C was determined for the labelled sequence in the absence of ligand (Fig. 2D). This value is very close to that measured by circular dichroism (Fig. 2A). In the presence of 2 equivalents of Phen-DC3 the melting temperature of the G4 increased by 7.5 °C (Fig. 2D), in agreement with the circular dichroism melting experiments (Fig. S1B,C). Again, these results strongly argue in favour of the formation of a G4 structure by the G-rich sequence HCV110-131, which is stabilized by Phen-DC3. Then, we performed the same assay in the presence of 15 or 50 equivalents of a double-stranded DNA competitor (ds26) (Fig. 2D,F). As expected in this competition experiment, Phen-DC3 did not bind to the double-stranded DNA competitor since the T_1/2_ was not affected by increasing concentrations of this duplex (Fig. 2D,F). The melting profile of ds26 DNA duplex and HCV-110-131 are provided in Fig. S3. The melting temperatures of ds26 DNA duplex (≈72 °C) is higher than the one of HCV-110-131 (≈60 °C).

Finally, the HCV107-151 RNA hairpin sequence (Fig. S2) was also used as a competitor in the assay. This RNA corresponds to the nucleotides 107 to 151 and allows the formation of a stem loop structure surrounding the G-rich sequence. HCV107-151 did not compete with HCV110-131 for the binding to Phen-DC3 (Fig. 2E,F). Hence, Phen-DC3 compound does not bind the HCV110-131 G-rich sequence when embedded in a stem loop structure but only binds to this sequence when folded into a G4 structure. The melting profile of HCV107-151 (melting temperature around 68 °C) is shown in Fig. S3.

### RNA dependent RNA synthesis by HCV RdRp is affected by RNA G4

**Figure 3:**
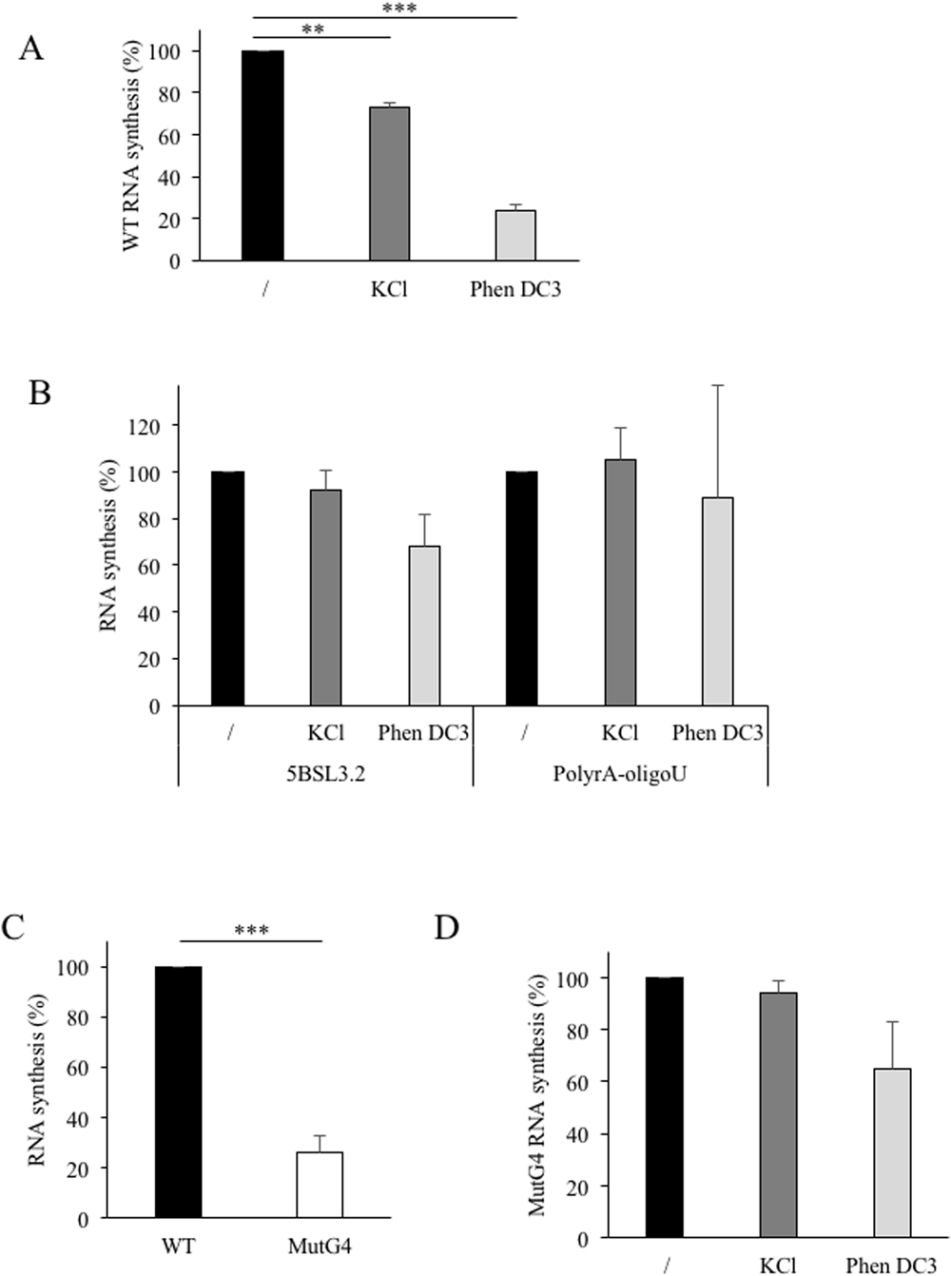
RNA-dependent RNA synthesis. (**A**) RNA synthesis on the WT template. The RNA corresponding to the 157 last nucleotides (Fig. 1A) was used as template in the RdRp assays with ^3^H-UTP labelled nucleotide. The amount of radioactivity incorporated into the nucleic acids synthesized was measured after 2 hours at 30 °C and expressed as the mean and standard deviation of the ratio to the WT. The experiment was performed in the presence of 40 mM NaCl, 40 mM KCl, or 40 mM NaCl plus 0.5 µM PhenDC3. (**B**) 5BSL3.2 sequence or PolyrA RNA in the presence of oligo(U) primer were used as templates in the conditions described in A. (**C**) Comparison of RNA synthesis on WT and mutated template (MutG4). (**D**) MutG4 template was used in RNA synthesis assay as described in A. For each conditions: n > 3 independent experiments in duplicate/error bars, mean ± SEM. Student test: no indication if p > 0.05; **p < 0.0005 and ***p < 0.00005.

Because this RNA G4 is located at the 3′-end of the (−)strand, which is the initiation site for the (+)strand replication by the RdRp, we investigated the putative role of this structure on RNA synthesis by HCV polymerase *in vitro*. To be active, the HCV polymerase requires the presence of cations. Only the NaCl and KCl were studied because the activity of the RdRp was inhibited by lithium cations (results not shown). The assay was first performed in the presence of 40 mM NaCl and corresponded to 100% activity. Figure 3A shows that when sodium cations were replaced by potassium cations known to stabilize G4 structures, the level of synthesis was decreased by 30%. In addition, the RNA synthesis was not significantly affected by the presence of KCl when using templates which did not contain any potential G4 such as the 5BSL3.2 RNA template (corresponding to the 3′-end of the (+)strand) or a primer-dependent elongation template (PolyrA-oligoU) (Fig. 3B).

To confirm the presence of a G-quadruplex at the 3′-end of the (−)RNA of HCV genome, the effect of Phen-DC3 on the RNA synthesis was analysed. Phen-DC3 was mixed with the RNA template before adding HCV RdRp, then the level of RNA synthesis was measured (Fig. 3A). Phen-DC3 hampered RNA synthesis by 76% when the WT substrate was used as template. The polymerase activity was not significantly affected in the presence of 0.5 µM of ligand on 5BSL3.2 template and remained unchanged using polyrA-oligoU template (Fig. 3B). These data suggest that the G4 ligand was able to interact with G4 sequence present at the 3′-end of the negative strand of HCV RNA.

To demonstrate these results, mutations were introduced in the G-rich sequence of the template to abolish G4 formation. RNA synthesis on the resulting MutG4 template was decreased in comparison to the WT sequence (Fig. 3C). However, the RNA synthesis was not affected by the presence of potassium (Fig. 3D). Furthermore, addition of Phen-DC3 did not affect significantly the RNA synthesis on MutG4 in comparison to the WT template.

Altogether, these results show that the RNA synthesis of the WT template was reduced in the presence of potassium or with Phen-DC3, which both stabilize the RNA G4 structure. Thus, in the context of the 157 last nucleotides of the HCV (−)strand, the G-rich sequence can be structured in a G-quadruplex and its presence affects RNA synthesis.

### G4 ligands inhibit HCV viral replication

**Figure 4:**
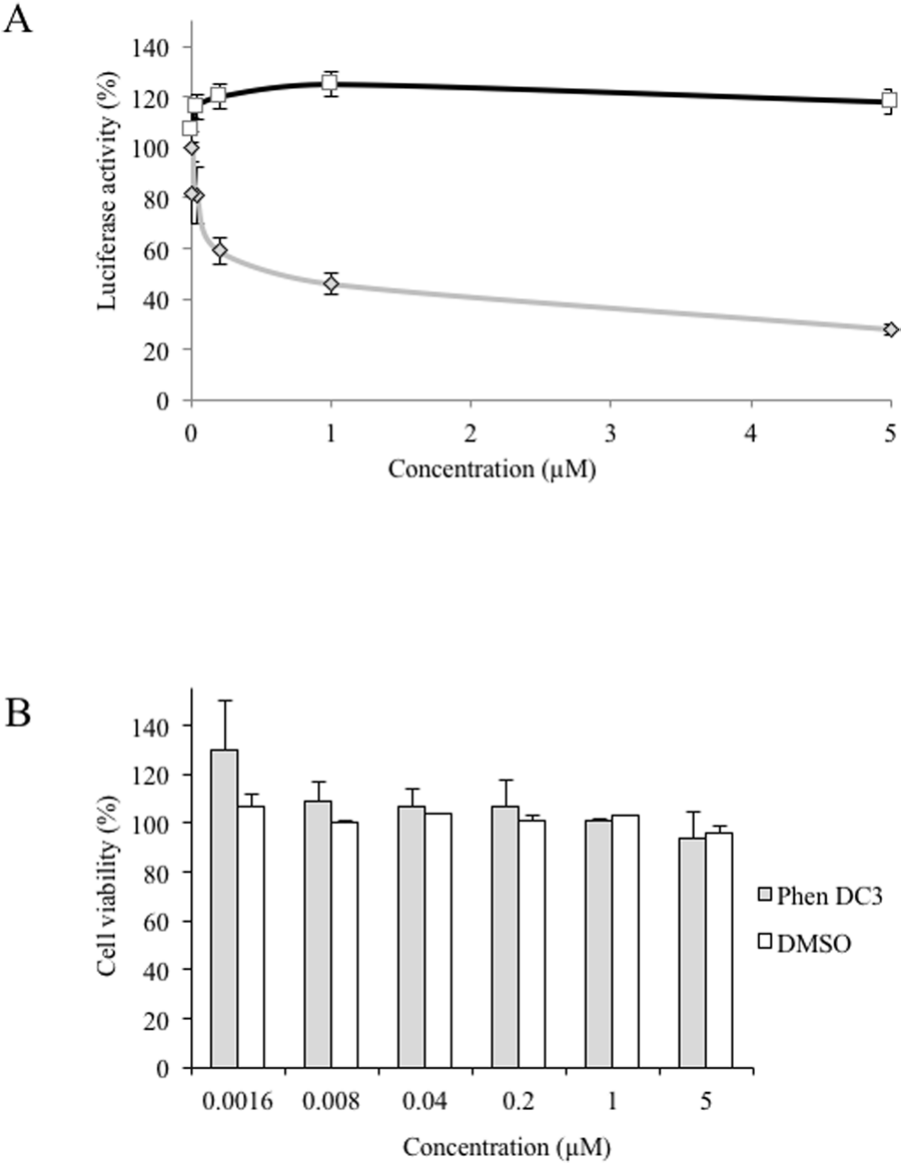
(**A**) G4 ligand activity on HCV replication. Serial dilutions of PhenDC3 (◊) or DMSO (□) were added on Huh7 cells which were then infected with LMTV-GLuc virus. The GLuc activity was measured after 48 hours of culture. The DMSO control corresponds to the DMSO percentage contained in each PhenDC3 concentration. (**B**) Cytotoxicity analysis of the ligand. Serial dilutions of PhenDC3 (◊) or DMSO (□) were added on Huh7 cells and analyse of cell viability was performed as described in Material and Methods. (n = 2 independent experiments in duplicate/error bars, mean ± SEM).

Because Phen-DC3 can bind the 3′-end of the intermediate strand of RNA replication, we wondered if this ligand could also inhibit the viral life cycle. To address this question, Huh7 cells were infected with the WT virus (LMTV) in the presence of increasing concentrations of Phen-DC3. The virus used in the infection assay express the Gaussia luciferase protein in frame with viral polyprotein such that the level of luciferase was directly related to viral replication^31^. As showed in Fig. 4A, GLuc expression was inhibited by 60% with 1 µM of Phen-DC3. The toxicity of Phen-DC3 on Huh7 cells was then measured. No effect on cell viability was observed up to 5 µM of ligand, the highest concentration that was assayed (Fig. 4B). This indicated that the viral replication was reduced in the presence of the G4 ligand in conditions where the cell viability was not affected.

## Discussion

The G4Hunter algorithm identified the G-rich sequence (nt 110–131) located in the IIy’ domain of the HCV 3′ (−)strand as a putative G4 forming sequence. Biophysical experiments confirmed that this RNA sequence could fold into a stable G4 structure. Mutations of this sequence were introduced within the last 157 nt to prevent G4 formation. Biochemical analysis confirmed these results by showing the effect of potassium and of the G4 ligand on the WT template but not the mutated sequence. We thus conclude that the G-rich sequence contained in the 157 last nucleotides of the HCV negative strand can form a G4 structure.

When we analysed the polymerase activity, the stabilisation of the structure by Phen-DC3 led to a decrease in the RNA synthesis. Yet the inhibition is observed on the template corresponding to the end of the HCV negative strand and not when using a synthetic template (polyrA) or a HCV RNA template without the G-rich sequence. This establishes that the polymerase enzymatic activity of the protein NS5B itself is not affected by the G4 ligand. The introduction of mutations in the G-rich sequence strongly affected the sensitivity to Phen-DC3 confirming the link between the inhibition and the formation of the G4 structure.

**Figure 5:**
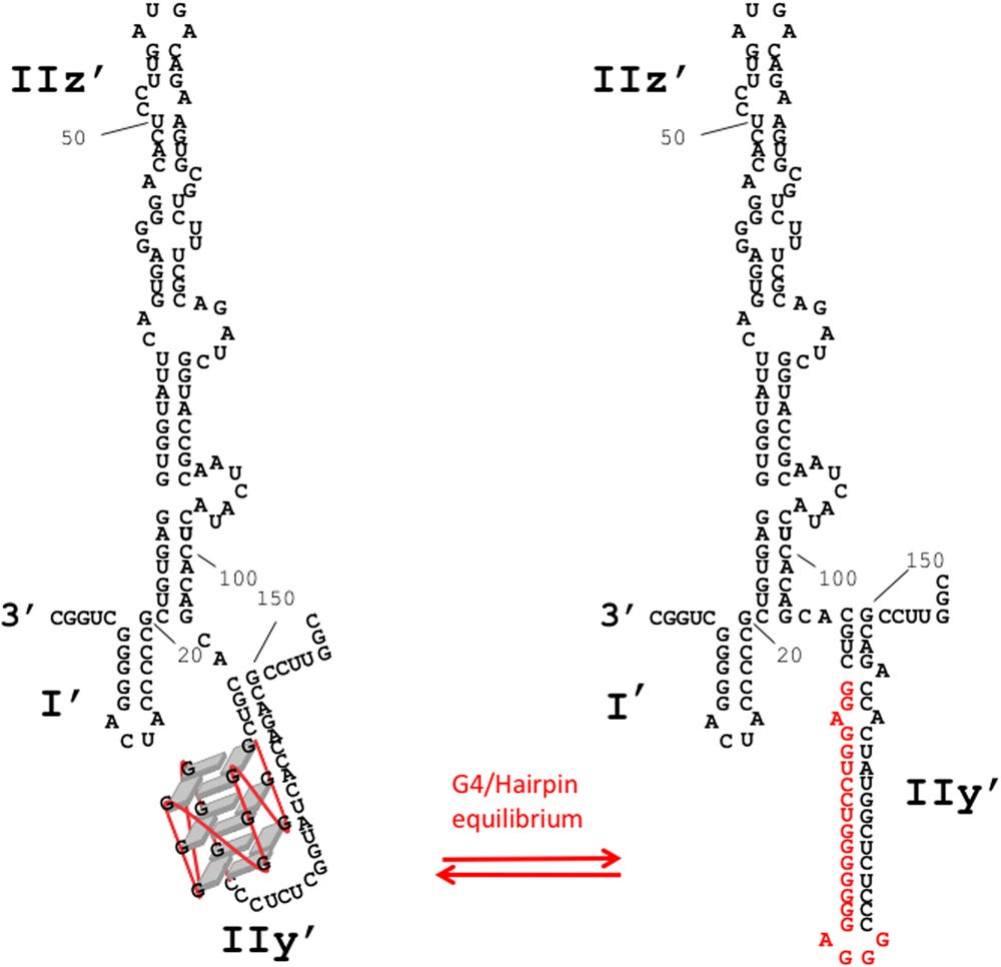
Schematic representation of alternative structures for an optimal RNA synthesis. The stem loop IIy’ could be folded either into a G-quadruplex conformation (left) or in Watson-Crick duplex conformation (right).

As mentioned in the Results section, the activity of the RdRp was reduced both when measured on (i) the MutG4 template where the G-rich sequence is mutated or (ii) on the WT template when the G4 is stabilized by the ligand or potassium. These surprising similar effects consisting in a decrease in the RNA synthesis raise the question of the requirement of an efficient dynamic between alternative structures for an optimal RNA synthesis (Fig. 5).

The structure published previously by Schuster *et al*. and Smith *et al*.^20,21^, showed a stem-loop in the IIy’ domain. The possibility of a G-quadruplex formation was not mentioned. In the structure proposed by Smith, G_114_ and G_115_ (in the middle of the IIy’ domain) were sensitive to RNAse T1 while the opposite nucleotides in the stem U_142_ and C_143_ were sensitive to RNase A. This suggests that these nucleotides may not be always engaged in Watson-Crick interactions. In addition, in the Schuster’s experiments, nucleotides G_121,122,123_ were slightly reactive to Pb^2+^ indicating that this region, at the bottom of the IIy’ domain, could be engaged in an alternative structure such as a G-quadruplex. These two models are compatible with our findings and suggest that the IIy’ domain in the (−)RNA strand of HCV could fold dynamically into mutually exclusive conformations, one being double-stranded as indicated by Smith and Schuster, the other organized into a G4 (Fig. 5). This structural dynamic could be essential for RdRp synthesis since mutation of G-rich sequence impeded its activity (Fig. 3B), while stabilisation of G4 by a known G4 ligand showed a similar effect. G-quadruplex is thus needed for RdRp synthesis but it has to be unstable enough for efficient RNA synthesis. How are these structures involved in the control of the virus replication remains to be investigated. Nevertheless, it is likely that the dynamic of this structure is needed for an efficient (+)strand RNA synthesis by the viral polymerase and this could constitute the basis of an efficient “quality control” for selecting the dynamically competent HCV (−)strand avoiding the replication of non-related or incomplete genomic RNA. We can expect RNA G4 to play an important role in the viral replication of RNA viruses as reported for DNA ones.

## Electronic supplementary material

Supplementary Information
