# Author Correction: RNA synthesis is modulated by G-quadruplex formation in Hepatitis C virus negative RNA strand

**DOI:** 10.1038/s41598-019-43445-7

**Published:** 2019-05-23

**Authors:** Chloé Jaubert, Amina Bedrat, Laura Bartolucci, Carmelo Di Primo, Michel Ventura, Jean-Louis Mergny, Samir Amrane, Marie-Line Andreola

**Affiliations:** 1 Univ Bordeaux, CNRS UMR5234, MFP laboratory, F-33000 Bordeaux, France; 2 Univ Bordeaux, ARNA laboratory, INSERM U1212, CNRS UMR 5320, IECB, F-33600 Pessac, France; 3 Institute of Biophysics, Academy of Sciences of the Czech Republic, 612 65 Brno, Czech Republic

Correction to: *Scientific Reports* 10.1038/s41598-018-26582-3, published online 25 May 2018

The original version of this Article contained errors in the spelling of the authors Chloé Jaubert, Amina Bedrat, Laura Bartolucci, Carmelo Di Primo, Michel Ventura, Jean-Louis Mergny, Samir Amrane and Marie-Line Andreola which were incorrectly given as Jaubert Chloé, Bedrat Amina, Bartolucci Laura, Di Primo Carmelo, Ventura Michel, Mergny Jean-Louis, Amrane Samir and Andreola Marie-Line respectively.

These errors have now been corrected in the PDF and HTML versions of the Article, and in the accompanying Supplementary Information File.

