# Supplementary material for: RNA synthesis is modulated by G-quadruplex formation in Hepatitis C virus negative RNA strand
Source: Sci Rep. 2018 May 25;8:8120. doi: 10.1038/s41598-018-26582-3 (PMC5970142; doi:10.1038/s41598-018-26582-3)
Supplement: Supplementary file 1 — Supplementary Information [file 41598_2018_26582_MOESM1_ESM.docx]

# RNA synthesis is modulated by G-quadruplex formation in Hepatitis C virus negative RNA strand

Chloé Jaubert^(a,*)^ ; Amina Bedrat^(b)^ ; Laura Bartolucci^(b)^ ; Carmelo Di Primo^(b)^ ; Michel Ventura^(a)^; Jean-Louis Mergny^(b,c)^ ; Samir Amrane^(b)^; Marie-Line Andreola^(a,*)^

# Authors list and affiliations

a- Univ Bordeaux, CNRS UMR5234, MFP laboratory, F-33000 Bordeaux, France.

b- Univ Bordeaux, ARNA laboratory, INSERM U1212, CNRS UMR 5320, IECB, F-33600 Pessac, France.

c- Institute of Biophysics, Academy of Sciences of the Czech Republic, 612 65 Brno, Czech Republic.

* To whom correspondence should be addressed

Email: [jaubert.chloe@gmail.com](mailto:jaubert.chloe@gmail.com) ; [marie-line.andreola@u-bordeaux.fr](mailto:michel.ventura@u-bordeaux.fr) Tel: +33 5 57 57 17 40 Fax: +33 5 57 57 17 66

# Supplementary information

**Figure S1**: **A**, Logo representation of the detected putative G4 sequence by G4Hunter algorithm. **B**, Circular dichroism spectra recorded in the presence of 10 mM lithium cacodylate buffer, pH 7.0, containing 10 mM of KCl. The HCV110-131 oligonucleotide concentration was 2.5 µM. PhenDC3 concentration was increased from 2.5 µM to 10 µM. **C**, Normalized circular dichroism melting profiles recorded at 263 nm in the presence of 10 mM lithium cacodylate buffer, pH 7.0, containing 10 mM of KCl. The HCV110-131 oligonucleotide concentration was 2.5 µM. PhenDC3 concentration was 2.5 µM and 5 µM.

**Figure S2**: **Biophysical caracterisation of the HCV45 stem loop structure.** **A**, Melting profiles recorded at 295 nm in the presence of 10mM of Lithium Cacodylate buffer pH7 supplemented with 100 mM of KCl, NaCl, LiCl. HCV107-151 Oligonucleotide concentrations was 2.5 µM. **B**, Thermal differential spectra recorded in the presence of 10mM of Lithium Cacodylate buffer pH7 supplemented with 100 mM of KCl, NaCl, LiCl. C. 1H 1D NMR spectra recorded in 20 mM potassium. **C**. 1H 1D NMR spectra recorded in 20 mM potassium phophate pH7 supplemented with 70 mM KCl. Oligonucleotide concentration was 100 µM. **D**. Schematic representation of HCV45 stem loop.

In the case of HCV110-154 oligonucleotide, the nature of the cation did not influence the stability of the structure (Figure S2A). At 260 nm, the same melting temperature of 75°C was observed in presence of Na+, K+ or Li+, while no G4 specific reversed transition was observed at 295 nm (Figure S2B). Along with the absence of a negative peak at 295 nm in the TDS spectra, these data show that HCV45 is not able to form a G4 structure (Figure S2B). Finally, the imino proton NMR spectrum of the HCV110-154 sequence presents a very different 1H 1D NMR signature with several imino peaks in the 10−11 ppm and 12-14 ppm regions. These resonances show the presence of canonical CG and AU base pairs as well as wobble GU base paires. HCV107-151 is therefore able to form a stem loop structure instead of a G-quadruplex structure.

**Figure S3**: CD spectrum (**A**) and CD melting curves recorded for HCV-110-131 and HCV107-151 RNAs and Ds26 DNA duplex sequence at 264 nm (**B**), 278 nm (**C**) and 250 nm (**D**). Oligonucleotides were dissolved at a concentration of 4µM in 10 mM lithium cacodylate buffer pH 7.2 containing 10 mM KCl and 10 mM LiCl.
